# Single Long Stents versus Overlapping Multiple Stents in the Management of Very Long Coronary Lesions: Comparisons of Procedures and Clinical Outcomes

**Published:** 2019-07

**Authors:** Alireza Amirzadegan, Mahdi Hasanabadi, Seyedmohammad Saadatagah, Mohsen Afarideh, Negar Omidi, Hassan Aghajani, Mohammad Alidoosti, Hamidreza Pourhosseini, Mojtaba Salarifar, Younes Nozari

**Affiliations:** *Tehran Heart Center, Tehran University of Medical Sciences, Tehran, Iran.*

**Keywords:** *Coronary artery diseases*, *Percutaneous coronary intervention*, *Stents*, *Drug-eluting stents*

## Abstract

**Background: **Different percutaneous coronary intervention (PCI) strategies, including the use of single long stents (SLSs) and overlapping multiple stents (OMSs), have been introduced to treat very long coronary lesions (VLCLs). The aim of this study was to compare procedural and long-term clinical outcomes between SLSs and OMSs in patients with VLCLs.

**Methods: **In this historical cohort study, 1709 patients who underwent PCI with the new generation of drug-eluting stents (length ≥38 mm) were stratified into the SLS [PROMUS /Resolute/XIENCE (PRX), (=38 mm), n=1121 (65.59%) and BioMime, (≥40 mm), n=124 (7.26%)] and OMS [(59.43±10.80 mm), n=464 (27.2%)] groups and followed up for 440.93±361.32 days. The study endpoints comprised immediate post-PCI outcomes, major adverse cardiovascular events (MACE), the patient-oriented composite endpoint (POCE), and the device-oriented composite endpoint (DOCE) at the long-term follow-up.

**Results:** The mean age of the patients was 59.28±10.60 years, and 69.6% of them were male. Flow grade 3 (P=0.296) and residual stenosis (P=0.533) were statistically similar between all the groups. A lower level of post-PCI troponin was observed in the BioMime group [14.52 (IQR_25%-75%_:10.44–22.42) ng/L; P=0.031] than in the PRX and OMS groups [18.63 (IQR_25%-75%_:10.51–34.02) ng/L and 18.96 (IQR_25%-75%_:11.17–35.34) ng/L; respectively]. Similarly, the PRX and BioMime groups received lower amounts of the contrast agent [206.29±49.15 mL and 208.06±55.23 mL; respectively] than did the OMS group [265.50±74.69 mL; P<0.001]. There were no statistically significant differences in the incidence of MACE [81 (7.2%), 7 (5.6%), and 28 (6.0%); P=0.603], the POCE [141 (12.6%), 13 (10.5%), and 54 (11.6%); P=0.731], and the DOCE [51 (4.5%), 4 (3.2%), and 21 (4.5%); P=0.791] between the PRX, BioMime, and OMS groups, respectively.

**Conclusion: **In the treatment of VLCLs, the SLS and OMSs appear to have similar clinical outcomes. BioMime ultra-long stents may have comparable results to PRX coronary stents.

## Introduction

Coronary artery disease is the leading cause of mortality worldwide.^1^^, ^^2^ Since it was first reported in 1977, percutaneous coronary intervention (PCI) has dramatically changed revascularization practice for patients with coronary artery disease.^3^^-^^4^ The considerable efficacy of drug-eluting stents in single discrete lesions has led to their application for longer and more complicated lesions.^5^^-^^8^ Despite the advancement in the stent platform, polymer, and techniques, intervention in very long coronary lesions (VLCLs) remains a major challenge.^9^ Different PCI strategies, including the use of single long stents (SLSs) and overlapping multiple stents (OMSs), have been introduced to treat VLCLs; nonetheless, the use of SLSs and OMSs is not yet a well-established method to ensure better outcomes and superiority. The significance of this issue becomes more evident when we know that the sole brand of ultra-long stents (≥40 mm) available in many countries, including Iran, is the BioMime (BioMime, Meril Life Sciences, India).^10^ Despite the low price of these stents by comparison with the more established brands such as the XIENCE, the PROMUS, and the Resolute, many physicians prefer to perform OMSs with 2 or even 3 of these well-regarded stents instead of an SLS by the BioMime because of concerns as regard the safety and the long-term outcome of the latter. The aim of this study was to compare procedural and long-term clinical outcomes between SLSs [PROMUS/Resolute/XIENCE (PRX) (=38 mm) or BioMime (40–48 mm)] and OMSs in patients with VLCLs.

## Methods

This is a historical cohort study conducted at Tehran Heart Center, Tehran, Iran. All the patients who received stent(s) with a minimum length of 38 mm were initially included and categorized into SLS and OMS groups. Subsequently, the SLS group was divided into 2 subgroups. 

**Figure 1:**
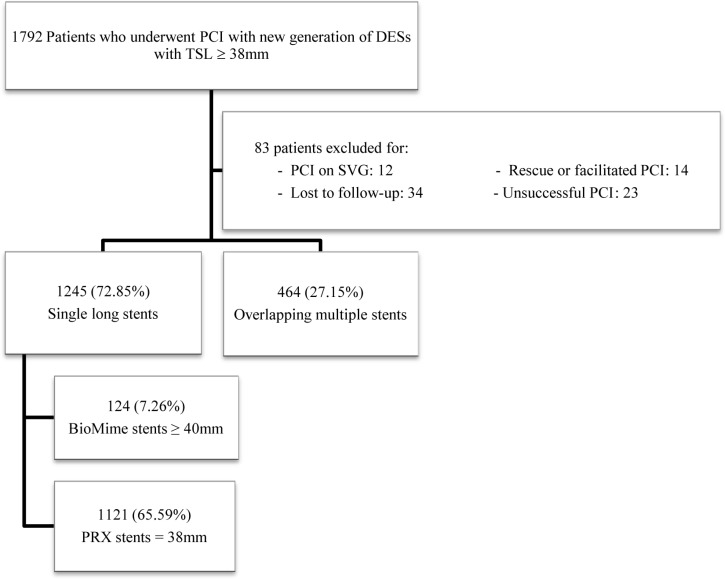
Flowchart of the patients enrolled in study

TSL, Total stent length; DES, Drug-eluting stent; PCI, Percutaneous coronary intervention; SVG, Saphenous venous graft; PRX, PROMUS/Resolute/XIENCE

The first subgroup was the PRX group, which consisted of patients with PROMUS, PROMUS Element, or PROMUS Element Plus stents (Boston Scientific, Natick, MA, USA); Resolute or Resolute Integrity stents (Medtronic Cardiovascular, Santa Rosa, California, USA); XIENCE, XIENCE V, XIENCE Prime, or XIENCE Xpedition stents (Abbott Vascular, Santa Clara, CA, USA); and BioMatrix or BioMatrix Flex stents (Biosensors Inc., Newport Beach, CA, USA). All these stents were 38 mm in length, the maximum length of these stents available in Iran at the time of the study. The initial for BioMatrix was not included in the PRX nomenclature due to the limited use (Figure 1). 

Patients who received bare-metal stents or those with the older generation of drug-eluting stents were excluded. The other exclusion criteria were facilitated or rescue PCI after the use of thrombolytic agents, PCI on saphenous vein grafts, and unsuccessful PCI due to the guide-wire passage failure or the stent passage failure. Patients who had incomplete data or PCI videos were also excluded. 

The second subgroup consisted of patients with BioMime stents with a minimum length of 40 mm.

All the recorded variables in this study were measured in accordance with the key data elements and definitions of the American College of Cardiology for measuring the clinical management and outcomes of patients with acute coronary syndromes.^   11 ^

The PCI procedures were performed by experienced academic interventional cardiologists according to the standard technique and access via the femoral or radial arteries. All the patients received standard of care and were pretreated appropriately with aspirin, clopidogrel, and atorvastatin in the context of previous medications. Heparin (100 U/kg) was injected at the time of catheterization. The entire study population received aspirin, a lifelong high-intensity statin regimen, and clopidogrel (75 mg/d) for at least 12 months after PCI, unless there was a contraindication. Other drugs were prescribed based on the recommended guidelines.^3^^, ^4 All the patients were actively engaged in regular visits at 1, 6, and 12 months’ follow-up, and additional visits were made if needed.

The 3 treatment groups were compared in terms of immediate procedural outcomes and long-term clinical outcomes. The immediate procedural success criteria were comprised of the thrombolysis in myocardial infarction (TIMI) flow grade, post-PCI residual stenosis, and the post-PCI troponin level. Periprocedural myocardial infarction was defined as a post-PCI troponin level of 5 times or more the upper limit of normal in patients who had normal baseline troponin.^12^ The amount of the contrast agent consumed was also compared between the treatment groups as a surrogate of radiation and the time of the procedure. 

The primary clinical endpoint was the incidence of major adverse cardiovascular events (MACE) as defined by cardiac death, nonfatal myocardial infarction, and revascularization, which comprised target vessel revascularization, target lesion revascularization, and coronary artery bypass grafting. The secondary endpoints were comprised of the patient-oriented composite endpoint (POCE), consisting of cardiac death, nonfatal myocardial infarction, coronary care unit admission due to unstable angina, target vessel revascularization, target lesion revascularization, and coronary artery bypass grafting; and the device-oriented composite endpoint (DOCE), consisting of cardiac death, target vessel revascularization, and target lesion revascularization. The date of event was considered to be the endpoint for the cases; for those without events, the last visit was considered to be the end of follow-up.

This study was conducted in accordance with the ethical standards of the Declaration of Helsinki. The study protocol was evaluated and approved by the Institutional Review Board of Tehran University of Medical Sciences, Tehran, Iran. Informed written consent was obtained from all patients on their first visit at Tehran Heart Center, Tehran, Iran.

The continuous variables were presented as the mean±standard deviations (SDs) if they assumed normal distributions and as medians (interquartile ranges) if they failed to assume normal distributions. The dichotomous variables were presented as numbers (percentages). The data were entered into the SPSS software, version 22.0 (SPSS Inc., Chicago, IL, USA), for analysis. Group comparisons for the categorical and continuous variables were performed using the χ^2 ^test, the Mann–Whitney test, the t-test, the Kruskal–Wallis test, and ANOVA, as appropriate. The univariate Kaplan–Meier and the multivariate Cox regression were used for survival analysis. The proportionality of hazards assumption was confirmed using the Schoenfeld residuals after fitting the Cox regression model as well as the observation of log-minus-log curves. The assumption of linearity was also verified by calculating martingale residuals. Stepwise adjustments were made for all potential confounders, including conventional cardiovascular risk factors and angiographic variables. Both crude and adjusted hazard ratios (HRs) were reported for the incidence of MACE, the POCE, and the DOCE. A P value of less than 0.05 was considered statistically significant in all the analyses.

## Results

A total of 1709 patients fulfilled the eligibility criteria and were enrolled in the final analysis (Figure 1). The mean age of the study population was 59.28±10.60 years, and 69.6% of them were male. There were 1121 (65.59%) patients in the PRX (=38 mm) group, 124 (7.26%) in the BioMime (≥40 mm) group, and 464 (27.15%) in the OMS group. The baseline demographic, clinical, laboratory, and echocardiographic characteristics of the study groups are presented in Table 1, which shows no significant differences between the 3 treatment groups. 

The types of lesions and procedure characteristics are outlined in Table 2. The procedural characteristics were similar between the SLS and OMS groups except for the prevalence of acute myocardial infarction, which was higher in the BioMime group (P<0.001). The OMS group had longer lesions and total stent length (55.42±10.78 mm and 59.43±10.80 mm, respectively) than did the PRX group (35.73±0.95 mm and 38.00±0.00 mm; P<0.001) and the BioMime group (42.20±2.73 mm and 44.23±2.51 mm; P<0.001). More additional balloons were used in the OMS group, which also underwent more PCI procedures in the myocardial infarction status than did the other 2 groups (P<0.001).

The immediate procedural and long-term clinical outcomes are presented in Table 3. The early procedural variables, including post-PCI TIMI (P=0.296) and residual stenosis (P=0.533), showed no significant differences between the 3 treatment groups. 

In the patients with a normal baseline troponin level, the average post-PCI troponin level was lower in the BioMime group [14.52 (IQR_25%-75%_: 10.44–22.42) ng/L] than in the PRX and OMS groups [18.63 (IQR_25%-75%_: 10.51–34.02) ng/L and 18.96 (IQR_25%-75%_: 11.17–35.34) ng/L; respectively, P=0.031]. The patients in the PRX group and the BioMime group (SLS group) received lower amounts of the contrast agent (206.29±49.15 mL and 208.06±55.23 mL; respectively; P<0.001) than did the patients in the OMS group (265.50±74.70 mL).

The mean follow-up period was 440.93±361.33 days, during which time the PRX, BioMime, and OMS groups represented with the incidence of MACE [81 (7.2%), 7 (5.6%), and 28 (6.0%); P=0.603], the POCE [141 (12.6%), 13 (10.5%), and 54 (11.6%); P=0.731], and the DOCE [51 (4.5%), 4 (3.2%), and 21 (4.5%); P=0.791], respectively. Moreover, no adverse events were statistically different between the 2 study groups (P>0.05). 

**Figure 2:**
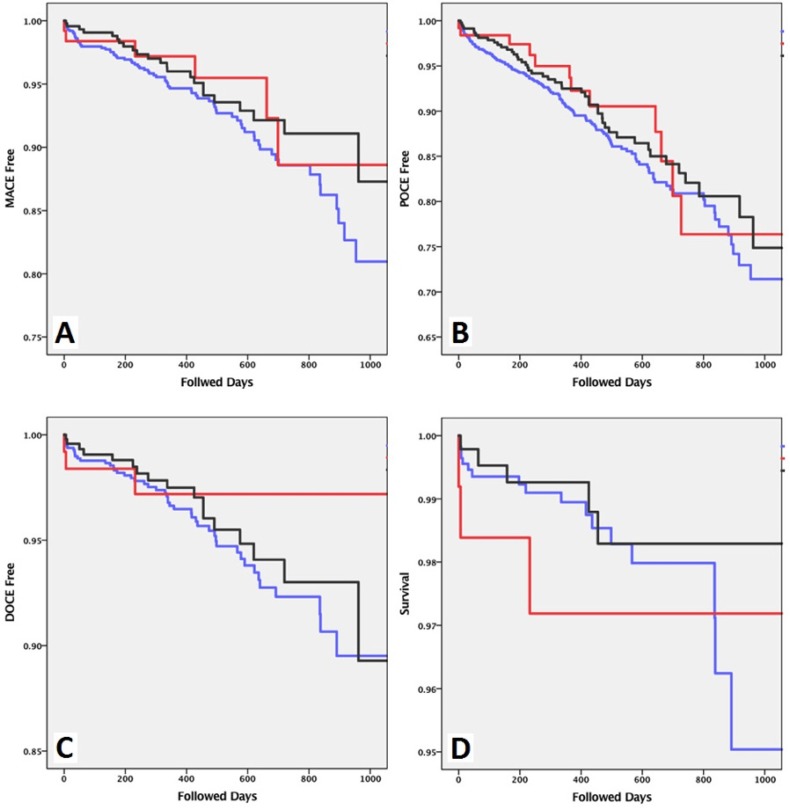
Kaplan-Meier plots presenting: A) MACE-free percentage (log-rank=0.533), B) POCE-free percentage (log-rank=0.599), C) DOCE-free percentage (log-rank=0.814), and D) survival (log-rank=0.507). Blue line represents the PRX group, the red line represents the patients with BioMime stents, and the black line represents the patients receiving OMSs.

MACE, Major adverse cardiovascular event; POCE, Patient-oriented composite endpoint; DOCE, Device-oriented composite endpoint; PRX, PROMUS/Resolute/XIENCE; OMS, Overlapping multiple stents

The Kaplan–Meier plots, showing the MACE-, POCE-, and DOCE-free periods and survival plots of the patients, as well as the corresponding Log-rank P values of 0.533, 0.599, 0.814, and 0.507, respectively, are presented in Figure 2.

 In Table 4, the PRX group was considered to be a reference group, and the HR of the incidence of MACE, the POCE, and the DOCE with the use of the BioMime or the OMS was calculated. Stepwise adjustment was performed for the conventional risk factors of coronary artery disease, the hallmarks of the index event, and lesion and procedure characteristics via the Cox regression model. As is shown in Table 3, in the fully adjusted model, the risks of MACE, the POCE, and the DOCE were not significantly different between the PRX and BioMime groups [HR=1.04 (95% CI: 0.41–2.65), P=0.923; HR=0.88 (95% CI: 0.44–1.78), P=0.730; and HR=1.03 (95% CI: 0.39–4.37), P=0.662] and also between the PRX and OMS groups [HR=0.87 (95% CI: 0.35–2.14), P=0.517; HR=0.84 (95% CI: 0.43–1.63), P=0.601; and HR=1.02 (95% CI: 0.35–2.96), P=0.962].

**Table 1:** Baseline clinical characteristics of the study population*

	**Single Long Stents**		**Overlapping Stents** **(n=464)**	**P**
	PRX = 38mm (n=1121)	BioMime ≥ 40mm (n=124)		
**Age (y)**	59.51±10.50	59.64±10.66	58.62±10.82	0.287
**Men**	781 (69.7)	89 (71.8)	299 (64.4)	0.081
**Hypertension **	638 (56.9)	76 (61.3)	288 (62.1)	0.134
**Diabetes **	334 (29.8)	33 (26.6)	156 (33.6)	0.196
** Diet**	37 (12.9)	5 (16.1)	16 (11.9)	0.811
** Oral agent**	191 (66.6)	23 (74.2)	310 (68.4)	0.498
** Insulin**	59 (20.6)	3 (9.7)	23 (17.0)	0.280
**Hypercholesterolemia **	269 (24.0)	22 (18.2)	111 (24.4)	0.329
**Hypertriglyceridemia**	308 (27.5)	42 (34.7)	134 (29.5)	0.220
**Family history of CAD **	206 (18.4)	18 (14.5)	96 (20.7)	0.254
**Smoking **				
** Current smokers**	283 (25.4)	28 (22.6)	111 (24.2)	0.730
** Former smokers**	166 (14.9)	26 (21.0)	57 (12.4)	0.068
**Opium**				
** Current users**	130 (11.6)	18 (14.5)	47 (10.1)	0.372
** Former users**	51 (4.5)	1 (0.8)	15 (3.2)	0.084
**History of CABG**	58 (5.2)	2 (1.6)	28 (6.1)	0.133
**History of PCI**	163 (14.6)	13 (10.5)	55 (11.9)	0.220
**BMI (kg/m2)**	27.95±4.57	28.14±4.17	27.97±4.59	0.913
**Creatinine (mg/dL)**	0.95±0.32	0.92±0.33	0.93±0.25	0.413
**Triglyceride (mg/dL)**	158.27±86.83	163.81±93.26	161.59±99.91	0.762
**Total cholesterol (mg/dL)**	168.01±44.14	162.36±40.84	168.13±46.85	0.414
**LDL-C (mg/dL)**	104.09±36.64	98.47±34.59	102.42±40.43	0.299
**HDL-C (mg/dL)**	38.76±10.31	36.69±10.11	39.94±10.29	0.072
**Ejection fraction (%)**	48.30±9.56	48.12±10.24	48.38±12.26	0.974

PRX, PROMUS/Resolute/XIENCE; CAD, Coronary artery disease; CABG, Coronary artery bypass graft surgery; PCI, Percutaneous coronary intervention; BMI, Body mass index; TG, Triglycerides; LDL-C, Low-density lipoprotein cholesterol; HDL-C, High-density lipoprotein cholesterol

* Data are presented as mean±SD or n (%).

**Table 2:** Lesion and procedure characteristics in the study population*

	Single Long Stents		Overlapping Stents (n=464)	P
	PRX=38mm (n=1121)	BioMime ≥40mm (n=124)		
Involved territory				
LAD	633 (56.6)	59 (47.6)	302 (65.1)	<0.001**
LCX	82 (7.3)	8 (6.5)	18 (3.9)	0.057
RCA	365 (32.6)	51 (41.1)	133 (28.7)	0.026***
Ramus	2 (0.2)	-	-	-
PDA-PLB	6 (0.5)	-	-	-
OM	31 (2.8)	6 (4.8)	11 (2.4)	0.333
Acute MI	245 (21.9)	50 (40.3)	80 (17.2)	<0.001**
Preprocedural stenosis (%)	90.0 [85.0-99.0]	90.0 [90.0-99.5]	90.0 [90.0 – 99.0]	0.478
Lesion length (mm)	35.73±0.95	42.20±2.73	55.42±10.78	<0.001***
Total stent length (mm)	38.00±0.00	44.23±2.51	59.43±10.80	<0.001***
Stent diameter**** (mm)	2.98±0.33	2.99±0.35	2.94±0.34	0.105
Pre-dilation	926 (82.6)	108 (87.1)	403 (86.9)	0.069
Post-dilation	961 (85.7)	104 (83.9)	384 (82.8)	0.312
Post-dilation pressure (atm)	18.62±2.95	18.85±3.40	18.88±2.95	0.312
Additional balloon	223 (19.9)	30 (24.2)	168 (36.2)	<0.001***
Pre-PCI TIMI flow				
0	181 (16.2)	28 (23.1)	73 (15.8)	0.151
1	59 (5.3)	7 (5.8)	35 (7.6)	0.208
2	82 (7.3)	10 (8.3)	41 (8.9)	0.576
3	798 (71.3)	76 (62.8)	314 (67.8)	0.072
Stent(s) type*****				
BioMime	-	124 (100)	18+59+1 (8.2)	-
BioMatrix	2 (0.2)		55+13+0 (7.3)	-
PROMUS	425 (37.9)		139+136+1 (29.1)	-
Resolute	195 (17.4)		36+38+2 (8.0)	-
XIENCE	499 (44.5)		216+218+15 (47.4)	-

PRX, PROMUS/Resolute/XIENCE; LAD, Left anterior descending; LCX, Left circumflex; RCA, Right coronary artery; PDA-PLB, Posterior descending artery-Posterior left ventricular branch; OM, Obtuse marginal; MI, Myocardial infarction; atm, Atmosphere; PCI, Percutaneous coronary intervention; TIMI, Thrombolysis in myocardial infarction

* Data are presented as n (%) or mean±SD, or median [IQR_25-75%_].

** Post-hoc analysis showed significant differences between the BioMime group and the other 2 subgroups.

*** Post-hoc analysis showed significant differences between all the subgroups.

**** Mean diameter of the stents used was reported in the OMS group.

***** Numbers placed after "+" refer to a stent used as a second or third stent.

**Table 3:** Immediate and late outcomes of the procedures*

	Single Long Stent		Overlap Stents (n=464)	P
	PRX=38mm (n=1121)	BioMime≥40mm (n=124)		
Immediate post-PCI outcome				
TIMI flow 3	948 (84.6)	97 (78.2)	378 (81.5)	0.296
Residual stenosis>30%	3 (0.3)	1 (0.8)	1 (0.2)	0.533
Post-PCI troponin ng/L	18.63 [10.51–34.02]	14.52 [10.44–22.42]	18.96 [11.17–35.34]	0.031**
Procedural MI***	111 (9.9)	5 (4.0)	60 (12.9)	0.011****
Contrast volume (mL)	206.29±49.15	208.06±55.23	265.50±74.69	<0.001*****
Long-term clinical outcomes				
MACE	81 (7.2)	7 (5.6)	28 (6.0)	0.603
POCE	141 (12.6)	13 (10.5)	54 (11.6)	0.731
DOCE	51 (4.5)	4 (3.2)	21 (4.5)	0.791
Cardiac death	17 (1.5)	2 (2.4)	5 (1.1)	0.525
Nonfatal MI	31 (2.8)	3 (2.4)	9 (1.9)	0.632
TVR	31 (2.8)	2 (1.6)	15 (3.2)	0.618
TLR	18 (1.6)	1 (0.8)	9 (1.9)	0.670
CABG	5 (0.4)	0	3 (0.6)	0.634
UA admitted in CCU	78 (7.0)	7 (5.6)	32 (6.9)	0.859

CABG, Coronary artery bypass graft; CCU, Coronary care unit; DOCE, Device-oriented composite endpoint; MACE, Major adverse cardiovascular event; MI, Myocardial infarction; PCI, Percutaneous coronary intervention; POCE, Patient-oriented composite endpoint; PRX, PROMUS/Resolute/XIENCE; TIMI, Thrombolysis in myocardial infarction; TLR, Target lesion revascularization; TVR, Target vessel revascularization; UA, Unstable angina

* Data are presented as n (%), mean±SD, or median [IQR_25-75%_].

** Post-hoc analysis showed significant differences between the BioMime group and the other 2 subgroups.

*** Defines a post-PCI troponin level 5 times or more than the upper limit of the normal range in those who had normal baseline troponin.

**** Post-hoc analysis showed significant differences between all the subgroups.

***** Post-hoc analysis showed significant differences between the OMS group and the other 2 subgroups.

**Table 4:** Cox regression modeling for the prognostic value of overlap stenting in predicting clinical outcomes*

	MACE		POCE		DOCE	
	HR (95% CI)	p	HR (95% CI)	p	HR (95% CI)	P
BioMime Model 0	0.79 (0.36-1.79)	0.547	0.83 (0.47-1.47)	0.524	0.72 (0.26-1.99)	0.527
BioMime Model 1	0.93 (0.42-2.03)	0.855	0.98 (0.55-1.74)	0.945	0.81 (0.29-2.26)	0.691
BioMime Model 2	0.79 (0.36-1.74)	0.555	0.90 (0.50-1.60)	0.718	0.71 (0.25-1.99)	0.512
BioMime Model 3	1.04 (0.41-2.65)	0.923	0.88 (0.44-1.78)	0.730	1.03 (0.39-4.37)	0.662
OMS Model 0	0.80 (0.52-1.23)	0.308	0.87 (0.63-1.19)	0.383	0.95 (0.57-1.56)	0.857
OMS Model 1	0.80 (0.52-1.27)	0.312	0.85 (0.61-1.17)	0.313	0.97 (0.58-1.61)	0.898
OMS Model 2	0.84 (0.54-1.31)	0.438	0.86 (0.62-1.20)	0.377	0.98 (0.58-1.66)	0.953
OMS Model 3	0.87 (0.35-2.14)	0.517	0.84 (0.43-1.63)	0.601	1.02 (0.35-2.96)	0.962

Model 0 is unadjusted.

Model 1 is adjusted for age, gender, smoking, opium abuse, diabetes, hypercholesterolemia, hypertension, and family history of coronary artery disease (CAD), body mass index, and serum creatinine (conventional risk factors of CAD).

Model 2 is additionally adjusted for percutaneous coronary intervention (PCI) in the acute coronary syndrome setting, pre-PCI stenosis, pre-PCI flow grade, history of coronary artery bypass graft surgery, history of PCI, and the ejection fraction (hallmarks of the index event).

Model 3 is additionally adjusted for the lesion length, the stent length, the mean stent diameter, the target vessel, and pre-dilation and post-dilation pressures (lesion and procedure characteristics).

MACE, Major adverse cardiovascular event; POCE, Patient-oriented composite endpoint; DOCE, Device-oriented composite endpoint; HR, Hazard ratio; OMS, Overlapping multiple stents

* PRX group was considered to be the reference group; and the hazard ratio (HR) of the incidence of MACE, the POCE, and the DOCE by using BioMime stents or OMSs was calculated.

## Discussion

The core findings from the present study indicated that the implementation of the single long BioMime stent had comparable clinical long-term results, including MACE, the DOCE, and the POCE, with overlap stents or PRX single stents. Moreover, the use of SLSs, as opposed to OMSs, conferred some advantages, especially in the term of cost, invasiveness of the procedure, the time of the procedure, the volume of the contrast agent, and radiation exposure. 

The application of PCI was prohibited for many years in patients with VLCLs due to a high risk of stent restenosis with bare-metal stents. However, recent studies have shown the safety of drug-eluting stents in long lesions^   13 ^ and even in the full metal jacket procedure.^14^^, ^^15^ Despite this scenario, the optimal application of PCI in patients with VLCLs has always been a source of great challenge. The use of OMSs has been shown to be associated with undesirable angiographic and clinical outcomes in some studies.^16^^, ^^17^ On the other hand, full lesion coverage with a stent is essential to prevent stent-edge restenosis,^15^^, ^^17^ which may be not achieved with SLSs, not least in the context of the unavailability of sufficient stent length. This situation warrants additional studies to assess the clinical outcomes of SLSs by comparison with OMSs for the treatment of VLCLs. Although several studies have reported adverse angiographic outcomes after the overlap of the first generation of drug-eluting or bare-metal stents,^5^^, ^^6^^, ^^8^ very limited data are available on the outcomes of new-generation drug-eluting stents.^7^^, ^^16^ 

In Iran, as is the case in many other countries, commercially available long stents are BioMatrix, XIENCE, PROMUS, and Resolute stents with an available maximum length of 38 mm, while BioMime stents are available in the lengths of 40, 44, and 48 mm. Therefore, many physicians prefer to perform single long stenting with 38-mm stents or to perform overlapping multiple stenting with 2 or even 3 of these well-regarded stents instead of single long stenting with BioMime stents because they are concerned about the safety and the long-term outcome of the latter. The preliminary study of BioMime stents presented favorable safety and performance in meriT-1, meriT-2, and meriT-3, in which the length of the stents used was between 13 and 40 mm.^10^^, ^^18^ More recently published small studies have demonstrated the feasibility and safety of BioMime stents in patients with VLCLs,^19^^, ^^20^  but this is a first report on the comparison of long-term safety and performance between BioMime stents and overlap stents or PRX single stents.

Apropos of the comparison between SLSs and OMSs in long lesions, similar studies were performed by De-Sheerder et al.^5^ (1998), Hoffman et al.^6^ (2002), and Pan et al.^8^ (2003), who reported findings similar to those in our study. Nonetheless, all the aforementioned studies included patients with lesion lengths of more than 20 mm and performed their comparisons using bare-metal stents. A More recent study by Mori et al.^7^ compared the angiographic and 1-year clinical outcomes between SLSs and OMSs in patients with lesions measuring between 30 and 38 mm in length. In their study, the authors used only XIENCE and PROMUS stents and showed that the rate of MACE and target lesion revascularization did not differ significantly between the 2 groups. As is evident, our study is superior to all the mentioned studies, in terms of the lesion and stent length, varied brands of newer generations of drug-eluting stents, and especially the number of patients with longer follow-up periods. 

The result of our study should not be interpreted as a recommendation for the use of SLSs in lieu of OMSs in all the patients with VLCLs. In point of fact, overlapping is obligatory in some cases. For example, 9.5% of our patients received OMSs longer than 60 mm and 5 patients received OMSs longer than 90 mm and obviously, these lesions could not be treated with SLSs. Additionally, in some cases, whereas SLSs will fail to pass the lesion, shorter stents are capable of making the passage, which necessitates overlapping.

The most significant limitation of this study is its observational design, meaning that the patients recruited were not randomized to receive either SLSs or OMSs. It should, however, be noted that our 3 groups had similar baseline conditions, thereby creating very little selection bias. The longer lesion lengths and longer stent lengths in the OMS group were simply unavoidable; nevertheless, we succeeded in making complete adjustments for these differences and also all other possible differences in the multivariate analysis. Because we excluded patients treated with bare-metal stents and older generations of drug-eluting stents, our findings should not be generalized to this category of patients. We performed clinical follow-ups instead of routine intracoronary assessments and conducted further angiographic assessments during the follow-up period only if clinical indications were present, which precluded an evaluation of the stent patency rate and the lumen loss. Although this study had an acceptable follow-up period, we believe that we could have achieved more robust conclusions had we had a longer follow-up.

Be that as it may, the present study is unique insofar as we restricted our inclusion criteria to patients with a minimum total stent length of 38 mm, which has not been studied before. Previous studies were performed on the use of bare-metal stents.^5^^, ^^8^ The exception is the study by Mori et al.,^7^ who used newer generations of drug-eluting stents; however, the stents utilized were limited to 2 brands of the XIENCE and the PROMUS. Our study is the first study of its kind to evaluate the safety and efficacy of BioMime stents with a minimum length of 40 mm in a relatively large cohort.

## Conclusion

The current study revealed that although the use of SLSs (either the BioMime or the PRX) and OMSs for the treatment of VLCLs yielded comparable clinical outcomes, the former conferred more cost-effectiveness, lower contrast administration, and less balloon use. Our observation further highlights the notion that BioMime ultra-long stents may be comparable to other well-established brands of coronary stents. Our results also showed that OMSs, if indeed indicated, could be a safe method.

## Notes:

***This paper should be cited as: ***Amirzadegan A, Hasanabadi M, Saadatagah S, Afarideh M, Omidi N, Aghajani H, Alidoosti M, Pourhosseini H, Salarifar M, Nozari Y. Single Long Stents versus Overlapping Multiple Stents in the Management of Very Long Coronary Lesions: Comparisons of Procedures and Clinical Outcomes. J Teh Univ Heart Ctr 2019;14(3):94-102.
